# Buildings Contemplated

**Published:** 1914-09-19

**Authors:** 


### Buildings Contemplated.

BRACEBRIDGE.—The Visiting Committee invite tenders for the enlargement of Bracebridge Asylum. Full particulars may be obtained of Mr. Frederick Parker, architect, The Square, Boston.

ILFORD.—The Ilford Urban District Council invite tenders for the erection of a phthisis sanatorium at the isolation hospital, Chadwell, Ilford. Full particulars may be obtained of Mr. H. Shaw, M.I.C.E., engineer and surveyor, Town Hall, Ilford.

IRVINE.—The Dean of Guild Court has passed plans for an infectious diseases hospital to be built by the Corporation between Kilwining Road and the north section of Irvine Moor. The cost is estimated at £5,000.

LEICESTER.—Tenders are invited for the erection of open-air class-rooms, additions, etc., at the Corporation Isolation Hospital, Anstey Lane, near Leicester. Quantities may be obtained of the Surveyor to the Education Committee, Town Hall.

MANCHESTER.—The Manchester Board of Guardians invite tenders for alterations to the cook-house, etc., at the infirmary of the Crumpsall Institution. Quantities may be obtained of Mr. A. J. Murgatroyd, architect, 23 Strutt Street, Manchester.

MANCHESTER.—The South Manchester Guardians have decided to proceed with the erection of a new children's hospital in Cavendish Road.

PAIGNTON.—The Local Government Board have held an inquiry into an application of the Urban District Council for permission to borrow £2,537 for an extension of the isolation hospital. Mr. C. O. Baines, the Council surveyor, has prepared the plans.

